# Most Hydrocarbonoclastic Bacteria in the Total Environment are Diazotrophic, which Highlights Their Value in the Bioremediation of Hydrocarbon Contaminants

**DOI:** 10.1264/jsme2.ME14090

**Published:** 2015-02-07

**Authors:** Narjes Dashti, Nedaa Ali, Mohamed Eliyas, Majida Khanafer, Naser A. Sorkhoh, Samir S. Radwan

**Affiliations:** 1 Microbiology Program, Department of Biological Sciences, Faculty of Science, Kuwait UniversityPO Box 5969, Safat 13060Kuwait

**Keywords:** bacteria, hydrocarbon bioremediation, oil pollution, oil utilization, nitrogen fixation

## Abstract

Eighty-two out of the 100 hydrocarbonoclastic bacterial species that have been already isolated from oil-contaminated Kuwaiti sites, characterized by 16S rRNA nucleotide sequencing, and preserved in our private culture collection, grew successfully in a mineral medium free of any nitrogenous compounds with oil vapor as the sole carbon source. Fifteen out of these 82 species were selected for further study based on the predominance of most of the isolates in their specific sites. All of these species tested positive for nitrogenase using the acetylene reduction reaction. They belonged to the genera *Agrobacterium*, *Sphingomonas*, and *Pseudomonas* from oily desert soil and *Nesiotobacter*, *Nitratireductor*, *Acinetobacter*, *Alcanivorax*, *Arthrobacter*, *Marinobacter*, *Pseudoalteromonas*, *Vibrio*, *Diatzia*, *Mycobacterium*, and *Microbacterium* from the Arabian/Persian Gulf water body. A PCR-DGGE-based sequencing analysis of *nifH* genes revealed the common occurrence of the corresponding genes among all the strains tested. The tested species also grew well and consumed crude oil effectively in NaNO_3_ -containing medium with and without nitrogen gas in the top space. On the other hand, these bacteria only grew and consumed crude oil in the NaNO_3_ -free medium when the top space gas contained nitrogen. We concluded that most hydrocarbonoclastic bacteria are diazotrophic, which allows for their wide distribution in the total environment. Therefore, these bacteria are useful for the cost-effective, environmentally friendly bioremediation of hydrocarbon contaminants.

Hydrocarbonoclastic microorganisms are involved in the mineralization of hydrocarbon pollutants in the total environment. The microbial utilization of such carbon-rich, but nitrogen-poor substrates necessitates the availability of nitrogenous compounds for the synthesis of cell materials such as proteins and nucleic acids. Nitrogen fertilizers have been shown to limit bioremediation in oily environments (5, 7, 8, 10, 11, 21, 22, 27). A previous study estimated that 60 mg N was needed for the microbiological consumption of 1 g hydrocarbons (9). Some environments, *e.g.* vegetation-poor, arid desert areas and seawater bodies, are naturally poor in nitrogenous compounds, as is the case in Kuwait and the whole Gulf area. However, oil spills enrich these environments with hydrocarbonoclastic bacteria (18, 19), which implies that indigenous hydrocarbonoclastic bacteria are nutritionally independent of exogenous nitrogenous sources due to their ability to fix atmospheric nitrogen. In 20 years of research on the microorganisms in oil-polluted areas of the Gulf, we identified numerous bacterial species with the coupled potential for hydrocarbon mineralization and nitrogen fixation in many habitats including desert soil, seawater, hypersaline areas, rhizospheres, phyllospheres, and air dust, (1, 2, 4, 6, 16, 17, 25). However, the relative frequencies of these bacteria have not yet been examined in the total environment.

The criteria for diazotrophy of the already studied isolates were growth on nitrogen-free media, the ability to reduce acetylene to ethylene, and, albeit very rarely, the possession of *nifH*-coding genes in bacterial genomes. These criteria have been used to confirm diazotrophic potential rather than actual *in situ* nitrogen fixation during growth on hydrocarbon substrates as sources of carbon and energy.

In the present study, we demonstrated that hydrocarbonoclastic bacteria isolated from all over Kuwait exhibited diazotrophic potential, as well as actual diazotrophic activity. Kuwait was suitable for this study because it is a small (approximately18,000 km^2^), but major oil-producing country. Furthermore, since it is a part of the semiarid zone of the globe, its environmental conditions are harsh; the maximum temperature during the long, dry summer may exceed 70°C while the minimum temperature in winter may reach 0°C. The vegetation-poor desert soil and Arabian Gulf water body are also poor in nitrogenous compounds.

## Materials and Methods

### Bacterial isolates

One hundred different species of oil-utilizing bacteria that have been deposited in our private culture collection were used in this study. The individual isolates were previously characterized by comparing the nucleotide sequences of their 16S rRNA genes with those of the closest relatives in the GenBank database. These species had been isolated from various pristine and oil-polluted Gulf habitats, including desert soil, the rhizospheres and phyllospheres of wild and cultivated plants, seawater, hypersaline areas, and air dust, on a mineral medium (24) with oil vapor as the sole source of carbon and energy. This mineral medium had the following composition (g L^−1^): 0.85 NaNO_3_ , 0.56 KH_2_ PO_4_ , 0.86 Na_2_ HPO_4_ , 0.17 K_2_ SO_4_ , 0.37 MgSO_4_ . 7H_2_ O, 0.7 CaCl_2_ . 2H_2_ O, and 2.5 mL of a trace element mixture consisting of (g L^−1^): 2.32 ZnSO_4_ , 1.78 MnSO_4_ , 0.56 H_3_ BO_3_ , 1.0 CuSO_4_ , 0.39 Na_2_ MoO_4_ , 0.42 CoCl_2_ , 0.66 KI, 1.0 EDTA, 0.4 FeSO_4_ , 0.004 NiCl_2_ at pH 7.0. Regarding isolations from sea water and hypersaline areas, 0.5 M and 2 M NaCl, respectively, were added to the medium. Details of the culture sources and methods for bacterial isolation and characterization have been described in our previous study (1).

### Assessment of diazotrophic potential

These 100 bacterial isolates were examined for “presumptive” nitrogen-fixation potential by testing their growth on the solid mineral medium (24) after removing NaNO_3_ , the sole nitrogen source available. An amount of 0.3% (w/v) crude oil (Light Kuwaiti crude), 0.3%, (w/v), was added as the sole source of carbon and energy. In order to confirm nitrogen-fixation potential, the 15 most dominant species in their habitats (oily desert soil and seawater) during initial plate-counting were tested for nitrogenase activity using the acetylene to ethylene reduction test (13) (the names of the bacterial species tested have been provided in the “Results” section). A cell suspension was prepared for each organism by mixing a loopful from a pure 48-h culture in 5 mL of the mineral nitrogen-free medium in a Hungate tube with a tight rubber cap. Using a gas-tight syringe, 10% of the head-space gas was replaced by acetylene. The medium used in this test contained 0.3% crude oil as the carbon and energy source. The tubes were incubated at 30°C for 12 d, and the head-space gas was analyzed for ethylene by gas-liquid chromatography (GLC) using a WCOT fused silica CP-SIL-5CB capillary column and temperature program 50–80°C in which the temperature was increased at 10°C min^−1^. Nitrogen was the carrier gas; the detector temperature was adjusted to 250°C and the injector temperature to 150°C. The appearance of an ethylene peak in the GLC profile indicated a positive test. Diazotrophy was confirmed by extracting the genomic DNA of the tested organism and amplifying the *nifH*-coding gene therein using the primers PolF (5′-TGCGAYCCSAARGCBGACTC-3′) and PolR (5′-ATSGCCATCATYTCRCCGGA-3′) (1, 15). The gene amplicons were subsequently separated and detected by gel electrophoresis. A loopful of the fresh culture was homogenized in 100 μL of PrepMan Ultra Sample Preparation Reagent (Applied Biosystems, USA) for genomic DNA extraction. The mixture was incubated in a water bath for 10 min at 100°C, then cooled for 2 min, and finally centrifuged at 14,000×*g* for 3 min to collect the DNA-containing supernatant. The PCR reaction mixture contained puReTaq Ready-To-Go PCR Beads (Amersham Biosciences, UK), 1 μL of DNA-containing supernatant, 1 μL each of the *nifH* primers (20 pmol μL^−1^) and the final volume was increased to 25 μL with molecular water (Sigma, UK). Amplification was achieved using the Veriti Thermal Cycler (Applied Biosystems) starting with initial denaturation for 5 min at 94°C, followed by 30 cycles of denaturation for 30 s at 94°C, annealing for 30 s at 58°C, and primer extension for 30 s at 72°C. The final extension was conducted for 5 min at 72°C. Gel bands carrying *nifH* DNA were cut out and the *nifH* fragments were reamplified with the same primers and then purified using the QIA quick PCR purification Kit (Applied Biosystems). Twenty nanograms of the DNA template was added to 2 μL of the BigDye version 3.1 terminator; 2 μL of the BigDye terminator 5× buffer, 1 μL of either PolF or PolR, and the final volume was increased to 10 μL with ultrapure water. The sequencing reaction was completed in the Veriti Thermal Cycler (Applied Biosystems) using a cycle of 96°C for 1 min, followed by 25 cycles of 1 min at 96°C, 5 s at 50°C, and 4 min at 60°C. Sequencing was performed in a 3130xl genetic analyzer (Applied Biosystems) with sequencing analysis software version 5.2 (Applied Biosystems). Sequences were subjected to a basic local alignment search tool analysis with the National Center for Biotechnology Information (NCBI; Bethesda, MD, USA) GenBank database (1). The classic nitrogen fixer *Klebsiella pneumonia* ATCC13883 was used as a positive control. The GenBank accession numbers are KP192902–KP192907.

Nitrogen fixation by the most frequently detected bacterial species (Table 1) was confirmed from a novel experiment designed for this purpose. Bacterial growth was measured in liquid mineral medium (24) aliquots containing NaNO_3_ and aliquots lacking this nitrogen source. Flask cultures were sealed and incubated at 30°C for 10 d. Growth in terms of optical density at 660 nm was measured at time zero and in 2-day intervals. Three parallel replicates were run for each reading and the mean values were calculated. Growth curves were plotted. The head-space gas of the cultures in some experiments was air (containing approximately 80% N_2_ ), but was completely substituted with a gas mixture consisting of 80% CO_2_ and 20% O_2_ (v/v) (*i.e.* no N_2_ gas was available) in other experiments. Tween 80, 0.3% (w/v), was used as the sole carbon and energy source in this experiment. This substrate had the advantage of being completely miscible with the aqueous medium. This was essential for subsequent optical density measurements. Another advantage was that Tween 80 contains long chain alkyl moieties, the carbon skeleton of aliphatic hydrocarbons. In this context, Tween 80 is the substrate utilizable by the unique group of the obligate hydrocarbonoclastic bacteria (OHCB) recently considered responsible for most of the natural removal of hydrocarbon contaminants in the marine ecosystem (28).

### Measurement of oil consumption

Crude oil consumption by individual bacterial species was measured quantitatively in batch cultures under the conditions specified above. One hundred-milliliter aliquots of the mineral medium (24) amended with 0.3% (w/v) crude oil (instead of Tween 80) were dispensed in 250-mL flasks. Each flask was inoculated with 0.25 mL of the bacterial cell suspension (a loopful of 48-h biomass in 5 mL sterile water). The cultures were sealed and incubated on an electric shaker at 120 rpm for 2 weeks. Residual crude oil was extracted with three successive 10-mL portions of pentane. The combined extract was increased to 35 mL using pentane, and 1 μL was analyzed by GLC. Regarding GLC, we used a Chrompack (NJ, USA) CP-9000 instrument equipped with a flame ionization detector and WCOT fused silica CP-SIL-5CB capillary column at a temperature of 10°C min^−1^. Nitrogen was the carrier gas; the detector temperature was 250°C and the injector temperature 150°C. The percentage loss of total peak areas in the GLC chromatograms based on the peak areas of the control samples (similarly prepared, but using previously autoclaved bacteria) was calculated as a quantitative measure of oil consumption. Three parallel replicates were run for each reading and the mean and standard deviation values were calculated.

## Results and Discussion

### Growth on nitrogen-free medium and possession of *nifH* genes

Eighty-two out of the 100 hydrocarbonoclastic bacterial species tested were capable of growing on the NaNO_3_ -free mineral medium with oil as the sole source of carbon and energy.

Table 1 shows the names of the 15 species selected for further study, as well as information related to their 16S rDNA sequencing. These species had their origin in Kuwaiti desert soil samples and seawater from the Arabian Gulf; they belonged to the bacterial subdivisions (classes); *Alphaproteobacteria*, *Betaproteobacteria*, *Gammaproteobacteria*, and *Actinobacteria*. The individual species exhibited 99 to 100% similarities in their 16S rDNA-sequences to their closest GenBank counterparts, with two species showing only 98 and 97% similarities.

**Fig. 1:**
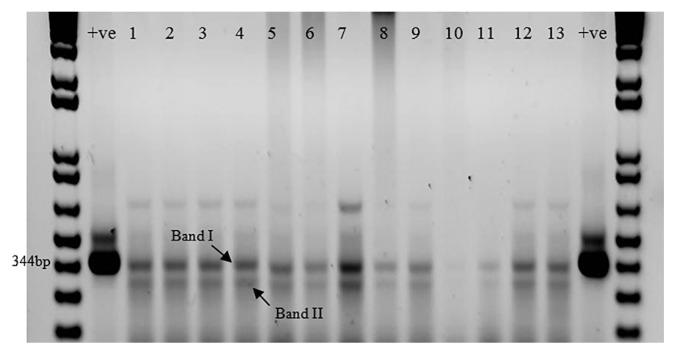
Typical gel electrophoresis bands of amplified *nifH* genes in the genomic DNA extract from the bacterial species tested. +ve, *Klebsiella pneumoniae* (Positive control); 1, *Microbacterium jejuense*; 2, *Nesiotobacter exalbescens*; 3, *Mycobacterium chlorophenolicum*; 4, *Dietzia maris*; 5, *Pseudoalteromonas tetraodonis*; 6, *Acinetobacter junii*; 7, *Alcanivorax dieselolei*; 8, *Nitratireductor aquibiodomus*; 9, *Pseudomonas stutzeri*; 10, *Vibrio fortis*; 11, *Agrobacterium tumefaciens*; 12, *Pigmentiphaga daeguensis*; 13, *Arthrobacter globiformis*. Band I and Band II for every species were cut, amplified, and sequenced, and the sequences obtained were then compared with the nearest GenBank matches (see Table 2).

These 15 bacterial species tested positive for nitrogenase, as revealed by the GLC-detection of ethylene peaks in their culture top space gases (GLC profiles not shown). The gel electrophoresis profiles in Fig. 1 confirmed that all the 13 species selected for analysis had *nifH* genes in their genomic DNA extracts.

The results of sequencing of the *nifH* gene bands in Fig. 1 are presented in Table 2. The bands of the 13 species tested revealed identical *nifH* gene sequences to those of heterotrophic bacteria in the GenBank database. The 3 species, *Sphingomonas dokdonensis*, *Marinobacter hydrocarbonoclasticus*, and *Pseudomonas xanthomonas* in Table 1 were not subjected to this analysis and, therefore, were absent in Table 2. *Sphingomonas dokdonensis* (1) and *Marinobacter* (*M. sedimentarum* and *M. flavimaris*) (3) were examined earlier in our laboratory for diazotrophy and tested positive. Furthermore, we only selected one of the two *Pseudomonas* sp. for this analysis. On the other hand, we subjected *Pigmentiphaga daeguensis* to this analysis (Table 2) even though it was not among the species in Table 1.

### Nitrogen gas was needed by hydrocarbonoclastic bacteria for growth and oil consumption in the absence of NaNO_3_

**Fig. 2:**
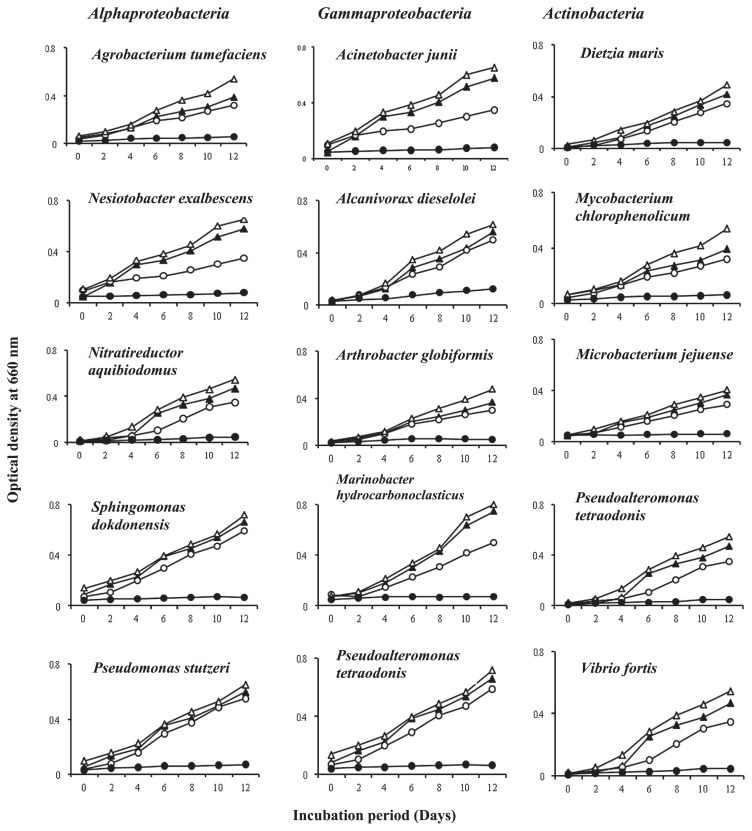
Growth curves of bacterial species in the presence and absence of NaNO_3_ and nitrogen gas. Open triangles, NaNO_3_ -containing medium with nitrogen gas; closed triangles, NaNO_3_ -containing medium without nitrogen gas; open circles, NaNO_3_ -free medium with nitrogen gas; closed circles, NaNO_3_ -free medium without nitrogen gas. There was only minimal growth, if any, in the NaNO_3_ -free medium with the top space free of nitrogen gas.

The growth curves in Fig. 2 showed that all the hydrocarbonoclastic strains tested grew well in the NaNO_3_ -containing mineral medium with Tween 80 (a hydrocarbon-like substrate) as the sole carbon and energy source. However, in the absence of NaNO_3_ , active growth only occurred when the top space of the cultures contained nitrogen gas.

**Fig. 3:**
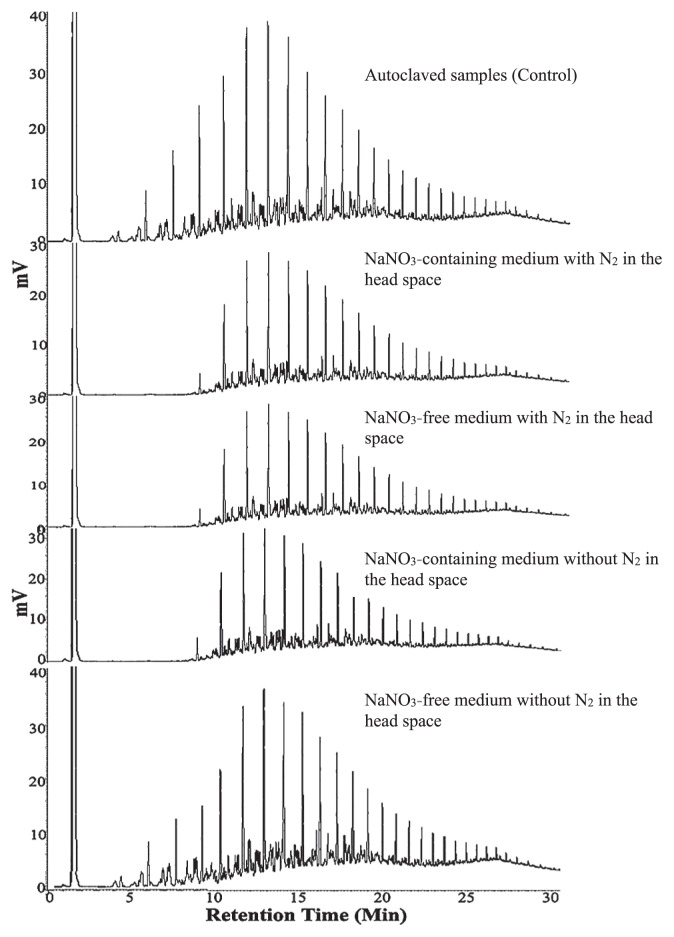
Typical GLC profiles showing the markedly effective consumption of crude oil by *Nesiotobacter exalbescens* in NaNO_3_ -free medium in the presence of nitrogen gas and poor consumption in its absence. Oil consumption was also markedly effective in the NaNO_3_ -containing medium with and without nitrogen gas in the head space. Weaker peaks indicate more effective consumption.

Table 3 shows the quantitative data of oil consumption by the individual bacterial species in the presence and absence of NaNO_3_ (in the medium) and nitrogen gas (in the head space). The results obtained clearly demonstrated that oil consumption was markedly effective in the NaNO_3_ -containing medium irrespective of whether nitrogen gas was present in the top space. However, the effective consumption of hydrocarbons only occurred in the NaNO_3_ -free medium when nitrogen gas was available in the top space, and was minimal in its absence. This was also clearly shown in Fig. 3, which presents the typical GLC profiles of residual hydrocarbons recovered from *Nesiotobacter exalbescens* growing in the presence and absence of NaNO_3_ and nitrogen gas for 2 weeks.

The novelty of this study is not only related to its methodology, but also to its major findings. Previous studies, mainly from our laboratory, described the combined potential of certain bacterial species for hydrocarbon utilization and nitrogen fixation. In addition to the habitats specified in the “Introduction” section, such bacteria have also been recorded in coastal mud-flats (4), epilithic biofilms (20), and hypersaline soils and waters (3).

One important result of the present study is that the *nifH* genes were detected unequivocally in all the environmentally predominant hydrocarbonoclastic bacterial species tested. Another novel result is that these bacterial species did not grow or exhibit any hydrocarbonoclastic activity in nitrogen-free media unless nitrogen gas was available. This result provides conclusive evidence for the ability of hydrocarbonoclastic bacteria to fix nitrogen *in situ*.

Microbiological nitrogen fixation is an activity that requires large amounts of energy. Therefore, when nitrogenous compounds and nitrogen gas are simultaneously available, diazotrophs prefer to utilize the former, which explains why nitrogen fertilizers enhance bacterial hydrocarbon bioremediation, as commonly reported, even though most hydrocarbonoclastic bacteria are diazotrophic. Based on their diazotrophy, such bacterial species have the ecological advantage of survival in environments extremely poor in (or free of) nitrogenous compounds.

It is experimentally challenging to unequivocally demonstrate diazotrophy in the 82 different bacterial species that gave positive presumptive tests. Therefore, we examined the most frequently detected species in desert soil and seawater in Kuwait. Most (12 species) of these species belonged to *Alphaproteobacteria* and *Gammaproteobacteria*, which was consistent with previous findings on hydrocarbonoclastic bacteria in contaminated sediments and seawater from a refinery harbor in the north of Tunisia (12). The occurrence, in all samples, of two gene bands instead of only one (Fig. 1) confirmed earlier findings that several versions of the *nifH* gene naturally occurred in the same diazotrophs (14, 15).

The results of this study are interesting from the view of recent studies in which hydrocarbons were shown to be utilized by conventional diazotrophic bacteria including *Rhizobium* (17), *Azotobacter* (26), and *Klebsiella* (1, 23). Therefore, we assumed that, in the course of early evolution, microorganisms that developed the ability to utilize nitrogen-poor substrates, such as oil, simultaneously developed the potential for nitrogen fixation in order to synthesize their nitrogenous cell materials (including proteins and nucleic acids). However, many molds and yeasts (eukaryotes that are incapable of nitrogen fixation) can also utilize hydrocarbons. Eukaryotic organisms appeared later in the course of evolution.

## Figures and Tables

**Table 1:** Oil-utilizing bacterial species from oil-contaminated sites in Kuwait selected for this study.

Strain	Sampling site	Subdivision	Nearest GenBank match	% identity	Bases compared	GenBank accession number
DSWAFI	Desert soil	*Alphaproteobacteria*	*Agrobacterium tumefaciens*	100	474	AB535688
SWDOH20	Seawater	*Alphaproteobacteria*	*Nesiotobacter exalbescens*	100	486	AF513441
SWSHR5	Seawater	*Alphaproteobacteria*	*Nitratireductor aquibiodomus*	100	477	EU440986
DSAMD18	Desert soil	*Alphaproteobacteria*	*Sphingomonas dokdonensis*	98	488	EU977661
SWFAH10	Seawater	*Gammaproteobacteria*	*Acinetobacter junii*	100	502	GU299535
SWSHR1	Seawater	*Gammaproteobacteria*	*Alcanivorax dieselolei*	97	513	HM584025
SWFAH1	Seawater	*Gammaproteobacteria*	*Arthrobacter globiformis*	100	475	FN908790
SWDOH2	Seawater	*Gammaproteobacteria*	*Marinobacter hydrocarbonoclasticus*	100	513	GQ901059
SWFAH8	Seawater	*Gammaproteobacteria*	*Pseudoalteromonas tetraodonis*	100	511	AB563179
DSSUB12	Desert soil	*Gammaproteobacteria*	*Pseudomonas stutzeri*	99	501	AJ270451
DSAMD14	Desert soil	*Gammaproteobacteria*	*Pseudomonas xanthomarina*	100	513	HM371425
SWSAL18	Seawater	*Gammaproteobacteria*	*Vibrio fortis*	99	521	EU419926
SWFAH3	Seawater	*Actinobacteria*	*Dietzia maris*	99	487	GQ870428
SWDOH21	Seawater	*Actinobacteria*	*Mycobacterium chlorophenolicum*	99	495	FJ544421
SWDOH9	Seawater	*Actinobacteria*	*Microbacterium jejuense*	100	488	EU419935

**Table 2:** Sequencing of gel bands I and II (in Fig. 1) carrying *nifH* genes in hydrocarbonoclastic bacterial genomes.

Bacterial species		Nearest GenBank Match					

		Band I	(%) Identity	Accession Number	Band II	(%) Identity	Accession Number
*Microbacterium jejuense* *Mycobacterium chlorophenolicum* *Alcanivorax dieselolei*	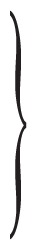	*Stenotrophomonas maltophilia* strain BJ01 dinitrogenase reductase (*nifH*) gene	100	KP192902	*Sinorhizobium meliloti* partial (*nifH*) gene for nitrogenase reductase, strain Gr79	100	KP192906
*Nesiotobacter exalbescens* *Acinetobacter junii* *Arthrobacter globiformis*	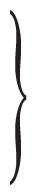	*Paenibacillus polymyxa* strain ISSDS-793 (*nifH*) gene	100	KP192903	*Sinorhizobium meliloti* partial (*nifH*) gene for nitrogenase reductase, strain Gr79	100	KP192906
*Vibrio fortis*		*Pseudomonas* sp. IPPW-3 nitrogenase iron protein (*nifH*) gene	91	KP192904	*Klebsiella variicola* strain 6A2 (*nifH*) gene	100	KP192907
*Pseudomonas stutzeri*		*Pseudomonas stutzeri* partial (*nifH*) gene for putative nitrogenase iron protein, strain Gr65	100	KP192905	*Klebsiella variicola* strain 6A2 (*nifH*) gene	100	KP192907
*Dietzia maris strain*		*Stenotrophomonas maltophilia* strain BJ01 dinitrogenase reductase (*nifH*) gene	100	KP192902	*Pseudomonas pseudoalcaligenes* strain CAN_BC2 dinitrogenase reductase Fe protein-like (*nifH*) gene	100	KP192908
*Nitratireductor aquibiodomus*		*Stenotrophomonas maltophilia* strain BJ01 dinitrogenase reductase (*nifH*) gene	100	KP192902	*Heliobacterium modesticaldum* (*nifH*) gene	100	KP192909
*Pseudoalteromonas tetraodonis*		*Pseudomonas stutzeri* partial (*nifH*) gene for nitrogenase iron protein, strain Gr65	100	KP192905	*Paenibacillus polymyxa* strain ISSDS-793 (*nifH*) gene	100	KP192903
*Agrobacterium tumefaciens*		*Pseudomonas* sp. IPPW-3 nitrogenase iron protein (*nifH*) gene	91	KP192904	Sequence failed		
Pigmentiphaga daeguensis*		Sequence failed			*Pseudomonas pseudoalcaligenes* strain CAN_BC2 dinitrogenase reductase Fe protein-like (*nifH*) gene	100	KP192908

* not included in Table 1.

**Table 3:** Crude oil-consumption by bacterial species in the presence and absence of NaNO_3_ in the medium and nitrogen gas in the culture top space.

Isolates	% Crude oil attenuation			

	N_2_ -containing head-space (air)		N_2_ -free head-space (CO_2_ +O_2_ )	
	
	NaNO_3_ -containing medium	NaNO_3_ -free medium	NaNO_3_ -containing medium	NaNO_3_ -free medium
*Agrobacterium tumefaciens*	32±1.5	26±1.3	31±1.5	4±0.1
*Nesiotobacter exalbescens*	40±1.9	33±1.3	41±2.0	7±0.1
*Nitratireductor aquibiodomus*	37±1.6	28±1.3	36±1.6	3±0.1
*Sphingomonas dokdonensis*	34±1.5	29±0.8	36±1.4	8±0.2
*Acinetobacter junii*	27±1.2	21±1.0	24±1.2	7±0.2
*Alcanivorax dieselolei*	28±1.4	24±1.1	29±1.9	7±0.2
*Arthrobacter globiformis*	34±1.7	27±1.3	32±1.5	8±0.2
*Marinobacter hydrocarbonoclasticus*	36±1.5	33±1.5	35±2.1	9±0.3
*Pseudoalteromonas tetraodonis*	35±1.6	28±1.3	38±1.8	7±0.1
*Pseudomonas stutzeri*	22±1.0	19±0.8	23±1.1	7±0.1
*Pseudomonas xanthomarina*	29±1.3	24±1.1	30±1.4	7±0.1
*Vibrio fortis*	37±1.6	29±1.4	38±2.3	8±0.3
*Dietzia maris*	29±1.4	27±0.9	22±1.1	8±0.2
*Mycobacterium chlorophenolicum*	33±1.5	27±1.2	37±1.5	6±0.1
*Microbacterium jejuense*	38±1.5	31±1.5	39±1.9	9±0.3

The mineral medium used was provided with 0.3% crude oil. Incubation was for 2 weeks.

Data were the means of 3 readings ± the standard deviation values. The very low consumption values in the right column were attributed to nitrogenous impurities in the medium that supported minimal bacterial growth and oil consumption.
